# Factors related to satisfaction with community-based home aging services in Shandong, China

**DOI:** 10.3389/fpubh.2024.1298669

**Published:** 2024-02-21

**Authors:** Yujing Wang, Qi Zhang, Laigang Huang, Fanshuo Zeng

**Affiliations:** Rehabilitation Medicine Center, The Second Hospital of Shandong University, Jinan, China

**Keywords:** healthcare integration, community care, satisfaction, aging, cost of aging

## Abstract

**Purpose:**

This study investigated the satisfaction of current community-based home care services and its factors in adults aged ≥60 years.

**Methods:**

Using stratified cluster random sampling, we surveyed 1,494 older adults in Jinan and Qingdao, Shandong province, between 2021 and 2023. The baseline and satisfaction surveys were designed by our research team, and the questionnaires were conducted in the form of structured interviews. *Kruskal-Wallis H-test* and *Logistic* regression analysis were used to explore the influencing factors of satisfaction.

**Results:**

The satisfaction was mainly affected by age (*p* = 0.007), marital status (*p* < 0.001), pre-retirement occupation (*p* = 0.003), economic source (*p* < 0.001), and mode of residence (*p* = 0.001) in the study of 1,494 older adults. Under the influence of multiple factors, the evaluation of older adults services, married [OR = 4.039, 95% CI: 1.176–13.877] were more inclined to be average, and their occupations were agriculture, forestry, animal husbandry, fishery, and water production workers [OR = 0. 237, 95% CI: 0.068–0.819] and production and transportation equipment operators and related personnel [OR = 0.153, 95% CI: 0.024–0.966] or [OR = 0.153, 95% CI: 0.029–0.820] tended to be more dissatisfied.

**Conclusion:**

The satisfaction level of community-based home care services is relatively high among older adults, and it is mainly affected by factors such as age, marital status, pre-retirement occupation, source of financial resources, and mode of residence. Addressing the emotional needs of older adults, lowering the cost of aging, and integrating health care and aging seamlessly are among the ongoing challenges that we need to tackle.

## Introduction

According to the statistics of China’s seventh population census in 2020, China’s total population has reached 1.4117.8 billion, the average annual population growth rate has dropped to 0.53%, and the proportion of older adults aged 60 and above in China has risen to 18.7%, an increase of 5.44% compared with the previous population census (1). China has entered a deeply aging society. In addition to the aggravating population aging, the increase in average life expectancy and the sharp rise in the incidence of disabling diseases such as cerebrovascular disease has led to an increase in the proportion of disability among older adults, further aggravating the burden of aging in China (2, 3). A tsunami of population aging is sweeping through China as the age structure of the population changes and the burden of disability increases. Compared with Japan and other aging societies, the gap between China’s economic development and its ability to cope with population aging policies remains more pronounced. Although China’s aging level is lower than Japan’s, it faces challenges such as a significantly older population base and a weak social security system (4, 5). China still has a long way to go in solving the problems that rapid aging may bring.

Under the influence of Confucianism, there is a unique model of old age in China. This model emphasizes “raising children to prevent old age,” i.e., family-centered, with children raising their aging parents. Traditional filial piety has become the cultural foundation for active aging in Chinese society (6). However, under the influence of the “family planning” policy, the size of family households in the seventh census is smaller than before, and young people in China are facing a situation where one person can support several older adults, bearing enormous financial pressure. And with urbanization and economic and social development, young people are often separated from their older adults and lack sufficient time to spend with them. Older adults are seen as a burden, which is not conducive to healthy aging. How young people balance “filial piety” and the reality of the pressure, but also needs the correct guidance of policies, so that the young labor force can create value without worry so that the older adults do not increase the burden of children and guilt.

In response to the economic and social turmoil brought about by the aging tsunami, China has introduced a series of policies in recent years to promote healthy aging. Yan et al. examined China’s policies from 1978 to 2019 using the Peking University Law Database (PKU Law), and China’s aging policy system has jumped from guaranteeing fundamental old-age conditions to focusing on the rights and health of the older population (7). Despite the continuous improvement of the basic old-age system, China’s old-age care industry still faces many problems, such as inadequate combination of medical care, low operability of intelligent devices for older adults, and the lack of old-age reserve talents (8, 9), so more and more governmental organizations are involved in the establishment of old-age policies. In 2021, multiple departments in China jointly issued the Action Plan for the Development of the Smart and Healthy Aging Industry (2021–2025). The text suggests that to promote the healthy growth of the aging industry, China’s tasks by 2025 mainly focus on enhancing the scientific and technological support capacity, improving the ability to supply products and services, highlighting the effectiveness of the pilot demonstration construction, optimizing and improving the industrial ecology, and promoting the integration of multi-industry integration and development (10). Taking this as our goal in recent years, we have driven the vigorous expansion of healthy aging by constantly adjusting and optimizing the structure of each aging industry.

Since the concept of “medical and nursing integration” was first proposed in 2013, China has continuously improved the policy of medical and nursing integration and issued Several Opinions on Deeply Promoting the Development of Medical and Nursing Integration in 2019 (11), which promotes the extension of China’s medical and nursing integration to the community and rural areas, to improve the efficiency and level of health care for the older adults. Through the integration of health care and social care, different from other pension methods that require leaving the familiar living environment, the older adults realized their wish of staying in the community and family through community home-based care; this approach greatly exerted the advantages of proximal nursing care, which not only reduced the cost of nursing care and alleviated the economic burden, but also respected the older adults’ autonomous choice and individual needs, and at the same time improved the older adults’ social participation and enhanced the sense of social belonging. Successful social participation requires a friendly physical and social environment (12). For the improvement of the physical environment, the state requires the continued acceleration of the construction of comprehensive service facilities in urban and rural communities, including the enhancement of rural flush toilets, the renovation of high-density overcrowded housing (13), the construction of day-care centers (9), the provision of “smart aging” services, the development of telemedicine, and “Internet+” customized housekeeping services, health care, and other aging services. Social environment improvement requires more inclusion of older adults, encouraging them to participate in social organizations, forming a mutual aid system (14, 15), and establishing a medical service system that benefits the older adults in the community. According to previous surveys on the needs of community-based nursing care, the demand for health care services was 48.15 and 52% (16, 17), respectively. The demand may differ by region, but the older population, being prone to chronic diseases, has a high need for health care. In 2022, to realize the reasonable distribution of medical resources in community older adults, the state adjusted the target of community medical and nursing services 2022 and listed older adults with incapacity, chronic diseases, and disabilities as the emphasis groups of medical and nursing services (18), which effectively meets the different health demands of the older adults in community older adult. With the gradual improvement of the physical and social environment, the sense of participation in family and social roles of the older adults aged at home in the community has been significantly improved, promoting the development of healthy aging.

Community-based home care services are not only a new type of older adult promoted by the policy of integrating medical care and nursing care but also an effective way of balancing the Chinese concepts of filial piety and “raising children for old age.” As the policies are continuously improved, the quality of community-based home care services has been enhanced. It is necessary to grasp the overall situation of community care and pay attention to the details of care services. What are the remaining shortcomings of community-based nursing care services in Shandong province under the policy promotion, and how should the nursing care services be adjusted based on these shortcomings? Therefore, a comprehensive satisfaction evaluation of community-based home care under the integration of healthcare and nursing and inquiry into its influencing factors are essential to promote the rapid development of healthy aging. In the seventh census, the proportion of older adults aged 60 and above in Shandong Province, China, reached 20.90% (1), far exceeding the national average. Facing the rapid population aging in Shandong Province, community-based nursing care can greatly alleviate the demand for nursing care; community-based nursing care exposed many problems as the community was the main unit of epidemic prevention and control, affected by the COVID-19 pandemic. Therefore, we conducted a questionnaire survey on 16 communities in two cities of Shandong Province, aiming to identify the shortcomings of community-based nursing care services and improve their service level.

## Methods

### Study sources

In this study, participants were selected in sixteen communities in Jinan and Qingdao cities of Shandong Province from October 2021 to November 2021 and from April 2023 to June 2023 using multi-stage stratified whole cluster random sampling. First, we randomly selected one county/district from each city administrative division. Second, we randomly selected two subdistricts from each county/district. Finally, we randomly selected four communities from each subdistrict. Randomly selected at least 100 people aged 60 years or older in each community. The inclusion criteria were (1) the age of the respondents should be ≥60 years old and (2) the respondents had received at least half a year and more of community-based home care services. The exclusion criteria were (1) unconsciousness and/or presence of cognitive dysfunctions and (2) those who refused to sign the informed consent form. To reduce the impact of COVID-19 on the interviews, we conducted the interviews at low-risk times. Due to the specific needs of community-based nursing care, the community opened the community dining hall for older adults at low-risk times to provide food services for them. We conducted most of our interviews in community dining halls, and used phone interviews for the older adults who were unable to visit the halls. To account for the diverse levels of comprehension and cultural backgrounds of the older adults, we employed trained interviewers who collected questionnaire data using face-to-face structured oral interviews, aiming to increase the response rate. The questionnaires filled out each day were scrutinized by the study leader.

The sample size for this study was calculated using the following formula:


$$n=\frac{\left[Z^{2}\times P\times \left(1-P\right)\right]}{d^{2}}$$

Equation *n* is the sample size, *Z* is the confidence level set at 1.96, *d* is the permissible margin of error set at 0.1*P*, *P* is the proportion of specific attributes in the overall population, and *P* is set at 0.5, the sample size was calculated to be 384 persons, and to minimize the sampling error of stratification, we increased the minimum sample size by 50%, that is, the required sample size was 576 participants.

Finally, we recruited a total of 1,630 participants, collected 1,494 valid questionnaires, with a response rate of 91.66%, reaching the required sample size.

### Study design

We integrated the previous research of related scholars and consulted professional questionnaires to design our own. We conducted a pre-survey, an expert review, and a reliability and validity test, and revised the questionnaire items accordingly. We then finalized the questionnaire that suited our study objectives. Among the questionnaires related to this study, the basic information questionnaire uniformly designed by the subject group was used, and its contents include: age, gender, marriage (unmarried, married, divorced, widowed), pre-retirement occupation of the respondents (civil servants, professional and technical personnel, clerical and related personnel, commercial and service personnel, agricultural, forestry, animal husbandry, fishery and water conservancy producers, production and transportation equipment operation related personnel, the military personnel, other practitioners who are inconvenient to classify, no occupation), education level (illiterate, elementary school, junior high school, high school/vocational high school/junior college, university and above), type of medical insurance (basic medical insurance for urban workers, basic medical insurance for urban and rural residents, poverty assistance, medical insurance, public funding, self-funding, and others), source of income (retirement pension/pension, children’s subsidies, financial support from relatives and friends, labor income, government subsidies, others), current mode of residence (living alone, living with spouse/partner, living with children, living with spouse and children, living with nanny/carer, others), number of children (none, 1, 2, 3, and above), number of times children visit (once a day, once a week, once a half-month, once a month, once every 3 months, once every half-year, once every 1 year and above), average monthly income (below 1,000 yuan, 1,001–3,000 yuan, 3,001–5,000 yuan, 5,001 and above), and satisfaction with community-based home care services (dissatisfied, average, satisfied). To ensure the authenticity and reliability of data from the research, we asked the community staff to recuse themselves and conduct the survey with anonymous structured interviews.

### Statistical analysis

Statistics were analyzed using IBM SPSS (V25) software. To accurately analyze the satisfaction of the older population with the current enjoyment of community-based home care services, this study assigns the value of “dissatisfied” as “1 point,” “general” as “2 points,” “satisfied,” and “3 points,” respectively. Count data were recorded as proportions (%). For continuous data, mean ± standard deviation (*X̅* ± *S*) was used if the data followed a normal distribution, and median (P25, P75) was used if the data were skewed. The data in this study showed skewed distribution, so the *Kruskal-Wallis H-test* was performed to analyze the differences between the different factors on the satisfaction of the community home care services, and the *post hoc* pairwise test was performed to adjust the significance value by *Bonferroni* correction to obtain the results of the differences within the group. After screening the independent variables, we performed a parallelism test and obtained *p* = 0.003, indicating that the data did not meet the parallelism assumption. We also checked for multicollinearity and found that the VIF values of all independent variables were less than 5, suggesting that there was no serious multicollinearity among them. Therefore, we used “dissatisfied” as the reference category and conducted a multinomial unordered logistic regression to examine the effects of specific factors on satisfaction (19). *p* < 0.05 was considered statistically significant.

## Results

### Socio-demographic characteristics

The study surveyed 1,630 older adults, excluding those with unclear information and those who did not fulfill the requirements, 1,494 valid questionnaires were recovered, with a response rate of 91.66%. The age distribution of the included 1,494 older adults was 74.00 (67.00, 82.00) years. The general information on older adults is summarized in Table 1.

**Table 1:** The demographic characteristics of the participants (*n* = 1,494).

Characteristics	Categorization	*N* (%)
Gender	Male	678 (45.4)
	Female	816 (54.6)
Marriage	Unmarried	145 (9.7)
	Married	962 (64.4)
	Divorced	22 (1.5)
	Widowed	365 (24.4)
Education level	Illiterate	359 (24.0)
	Elementary school	433 (29.0)
	Junior high school	318 (21.3)
	High school/vocational high school/junior college	292 (19.5)
	University and above	92 (6.2)
Pre-retirement occupation	Civil servants	266 (17.8)
	Professional and technical personnel	202 (13.5)
	Clerical and related personnel	30 (2.0)
	Commercial and service personnel	119 (8.0)
	Agricultural, forestry, animal husbandry, fishery and water conservancy producers	465 (31.1)
	Production and transportation equipment operation related personnel	51 (3.4)
	The military personnel	9 (0.6)
	Other practitioners	144 (9.6)
	No occupation	208 (13.9)
Current mode of residence	Living alone	258 (17.3)
	Living with spouse/partner	540 (36.1)
	Living with children	149 (10.0)
	Living with spouse and children	168 (11.2)
	Living with nanny/carer	206 (13.8)
	Others	173 (11.6)
Type of medical insurance	Basic medical insurance for urban workers	534 (35.7)
	Basic medical insurance for urban and rural residents	719 (48.1)
	Poverty assistance	148 (9.9)
	Medical insurance	10 (0.7)
	Public funding	10 (0.7)
	Self-funding	24 (1.6)
	Others	49 (3.3)

### Older adults’ satisfaction with the community-based home care services

Analyzing the overall satisfaction with the current community-based home care services, 42 people, or 2.8%, were “dissatisfied,” 171 people, or 11.5%, were “average,” and 1,268 people, or 85.6%, were “satisfied.” 1,268, accounting for 85.6%. The degree of satisfaction is assigned 1–3 points (Table 2), and the overall satisfaction distribution was 3.0 (3.0, 3.0). In general, the current older population has a relatively high level of satisfaction with community home care services.

**Table 2:** The satisfaction level of the community nursing care services among the 1,494 participants.

Satisfaction	Frequency (persons)	Percentage (%)
Dissatisfied (1 point)	42	2.8
Average (2 points)	171	11.5
Satisfied (3 points)	1,268	85.6
Absent	13	

### Factors affecting satisfaction with community-based home older adult services

As shown by the quartiles in the Table 3, the satisfaction scores for each factor are fairly uniform, with no significant outliers, suggesting that the community-based nursing care services in China have a stable level of overall satisfaction. Univariate analysis of the questionnaire results revealed that age (*p* = 0.007), marital status (*p* < 0.001), pre-retirement occupation (*p* = 0.003), economic source (*p* < 0.001), and mode of residence (*p* = 0.001) were statistically significant in affecting satisfaction with community home-based aging services. On the other hand, gender (*p* = 0.389), education level (*p* = 0.325), number of children (*p* = 0.185) and number of children’s visits (*p* = 0.510), average monthly income (*p* = 0.444), and type of health insurance (*p* = 0.202) did not show any statistically significant differences, which had little impact on satisfaction (Table 3).

**Table 3:** Identified the factors affecting the satisfaction of the older age population.

Categorization	Specified variable	Satisfaction scores *M* (*P*_25_, *P*_75_)	*p*-value^a^
Gender	Male	3.00 (3.00, 3.00)	0.389
	Female	3.00 (3.00, 3.00)	
Age	60–69 years	3.00 (3.00, 3.00)	0.007
	70–79 years	3.00 (3.00, 3.00)	
	80–89 years old	3.00 (3.00, 3.00)	
	Above 90 years old	3.00 (3.00, 3.00)	
Marriage	Unmarried	3.00 (3.00, 3.00)	<0.001
	Married	3.00 (3.00, 3.00)	
	Divorced	3.00 (3.00, 3.00)	
	Widowed	3.00 (3.00, 3.00)	
Education level	Illiterate	3.00 (3.00, 3.00)	0.325
	Elementary school	3.00 (3.00, 3.00)	
	Junior high school	3.00 (3.00, 3.00)	
	High school/vocational high school/junior college	3.00 (3.00, 3.00)	
	University and above	3.00 (3.00, 3.00)	
Pre-retirement occupation	Civil servants	3.00 (3.00, 3.00)	0.003
	Professional and technical personnel	3.00 (3.00, 3.00)	
	Clerical and related personnel	3.00 (3.00, 3.00)	
	Commercial and service personnel	3.00 (3.00, 3.00)	
	Agricultural, forestry, animal husbandry, fishery and water conservancy producers	3.00 (3.00, 3.00)	
	Production and transportation equipment operation related personnel	2.00 (3.00, 3.00)	
	The military personnel	3.00 (3.00, 3.00)	
	Other practitioners	3.00 (3.00, 3.00)	
	No occupation	3.00 (3.00, 3.00)	
Source of income	Retirement pension/pension	3.00 (3.00, 3.00)	<0.001
	Children’s subsidies	3.00 (3.00, 3.00)	
	Financial support from relatives and friends	3.00 (3.00, 3.00)	
	Labor income	3.00 (3.00, 3.00)	
	Government subsidies	3.00 (3.00, 3.00)	
	Others	3.00 (3.00, 3.00)	
Current mode of residence	Living alone	3.00 (3.00, 3.00)	0.001
	Living with spouse/partner	3.00 (3.00, 3.00)	
	Living with children	3.00 (3.00, 3.00)	
	Living with spouse and children	3.00 (3.00, 3.00)	
	Living with nanny/carer	3.00 (3.00, 3.00)	
	Others	3.00 (3.00, 3.00)	
Number of children	None	3.00 (3.00, 3.00)	0.185
	1 person	3.00 (3.00, 3.00)	
	2 persons	3.00 (3.00, 3.00)	
	3 persons and above	3.00 (3.00, 3.00)	
Number of times children visit	Once a day	3.00 (3.00, 3.00)	0.510
	Once a week	3.00 (3.00, 3.00)	
	Once a half-month	3.00 (3.00, 3.00)	
	Once a month	3.00 (3.00, 3.00)	
	Once every 3 months	3.00 (3.00, 3.00)	
	Once every half-year	3.00 (3.00, 3.00)	
	Once every one year and above	3.00 (3.00, 3.00)	
Average monthly income	Below 1,000 yuan	3.00 (3.00, 3.00)	0.444
	1,001–3,000 yuan	3.00 (3.00, 3.00)	
	3,001–5,000 yuan	3.00 (3.00, 3.00)	
	5,001 and above	3.00 (3.00, 3.00)	
Type of medical insurance	Basic medical insurance for urban workers	3.00 (3.00, 3.00)	0.202
	Basic medical insurance for urban and rural residents	3.00 (3.00, 3.00)	
	Poverty assistance	3.00 (3.00, 3.00)	
	Medical insurance	3.00 (3.00, 3.00)	
	Public funding	2.00 (3.00, 3.00)	
	Self-funding	2.00 (3.00, 3.00)	
	Others	3.00 (3.00, 3.00)	

a The Kruskal-Wallis H test was employed to evaluate the results.

We identified between-group differences in age, marital status, occupation before retirement, primary source of income, and lifestyle with *post hoc* multiple pairwise tests and *Bonferroni* corrections to adjust significance (Supplementary Tables S1–S5). In the age group *post hoc* tests, no significant differences were seen between groups after adjusting for salience with the *Bonferroni* correction. Widowhood was statistically different from unmarried and married (*p* = 0.001, *p* = 0.005, respectively). There was a statistical difference in attitudes toward satisfaction with old age between the older adults who were predominantly professional and technical personnel and those who were not, production and transportation equipment operators and related personnel (*p* = 0.022, *p* = 0.006, respectively). There was also a significant difference in financial sources between those receiving government benefits and those receiving pensions and subsidies from their children (*p* < 0.001, *p* = 0.001, respectively).

### Regression analysis of the satisfaction of community-based home care services

With the satisfaction of the current community home care service as the dependent variable, the disordered multinomial *Logistic* regression analysis was used to analyze the different factors. Age, marital status, pre-retirement occupation, economic source, and mode of residence, which were statistically significant differences in the single-factor analysis, were imported into the model. The Nagelkerke *R*^2^ indicates that the model explains 14.7% of the variation in satisfaction with community-based nursing care. The Omnibus Tests of Model Coefficients yields a chi-square of 141.496, *p* < 0.001, indicating that the model (with explanatory variables) fits well. According to the analysis estimate, the overall accuracy of the correct prediction probability was 85.6% (20–22). The results of the regression analysis showed that age, income source, and living arrangement did not have statistically significant differences. The statistical differences of marital status and occupation are shown in Table 4. According to the data analysis results, compared with the widowed group, the married older adults were more likely to choose “general” satisfaction (*p* = 0.027), and the widowed older adults were more likely to choose “dissatisfied”; Production personnel in agriculture, forestry, animal husbandry, fishery, and water conservancy (*p* = 0.023), production and transportation equipment operators and related personnel (*p* = 0.046) were more likely to be “dissatisfied” than those without occupation. The non-occupational group was more inclined to be “average” and “satisfied “(Table 4).

**Table 4:** The regression analysis of the effects of marital status and occupation on the satisfaction of community home-based nursing care services.

	Satisfaction: average			Satisfaction: satisfactory		
	*B*	*p*-value	OR (95%CI)	*B*	*p*-value	OR (95%CI)
Marriage						
Unmarried	0.278	0.711	1.321 (0.304, 5.748)	−0.414	0.536	0.661 (0.178, 2.449)
Married	1.396	0.027	4.039 (1.176, 13.877)	0.156	0.779	1.169 (0.394, 3.472)
Divorced	0.833	0.568	2.301 (0.132, 40.238)	0.534	0.669	1.705 (0.147, 19.749)
Widowed (contrast)						
Occupation						
Civil servants	−0.848	0.337	0.428 (0.076, 2.415)	−0.137	0.867	0.872 (0.177, 4.302)
Professional and technical personnel	14.463	0.990	1910633.595 (Absent)	15.467	0.989	5213692.161 (Absent)
Clerical and related personnel	13.719	0.993	907777.941 (Absent)	14.326	0.993	1665661.859 (Absent)
Commercial and service personnel	−1.448	0.073	0.235 (0.048, 1.142)	−0.783	0.287	0.457 (0.108, 1.932)
Agricultural, forestry, animal husbandry, fishery and water conservancy producers	−1.442	0.023	0.237 (0.068, 0.819)	−0.404	0.489	0.668 (0.213, 2.098)
Production and transportation equipment operation related personnel	−1.880	0.046	0.153 (0.024, 0.966)	−1.875	0.028	0.153 (0.029, 0.820)
The military personnel	14.507	0.998	1996274.988 (Absent)	15.171	0.998	3877386.452 (Absent)
Other practitioners	0.740	0.537	2.096 (0.201, 21.902)	1.351	0.242	3.862 (0.401, 37.159)
No occupation (contrast)						

We conducted a disordered multinomial Logistics regression analysis to assess the outcomes.

## Discussion

This questionnaire survey found that the current population of community-based home care services is more receptive and more satisfied with the service situation. The degree of satisfaction of older adults is mainly affected by factors such as age, marital status, pre-retirement occupation, source of income, and mode of residence.

The emotional needs of older adults play a crucial role in evaluating satisfaction. Of the data on age, marital status, and mode of residence of older adults, number of children, and number of visits by children, only age, marital status, and current resident manner significantly affected satisfaction. Although there was no statistically significant difference in satisfaction between age groups after Bonferroni correction, for the older adults over 80 years old, there will be professional and full-time personnel in the community to take care of the older adults according to the situation, the community pension policy for the older adults is more inclined, so in the case of the physically fit older adults, they can both receive professional personal care and can also enjoy their family life in the community. Due to the unique nature of community-based home care services, family-centered and community-supported nursing care can better meet the well-being of older adults, who do not need to adapt to a new living environment (23). Under the community home care model, older adults do not need to stay away from the family, can get away from the triviality of life during the day, have their own leisure time, and enjoy the happiness of their grandchildren at night. Previous studies have shown that the number of children and the frequency of their visits have a significant impact on the quality of institutional nursing care (24), while community-based home care is less affected by these factors, which is one of its advantages.

However, in the post-test results of marital status, the satisfaction of the widowed differs from that of the unmarried and married, respectively. A comprehensive analysis of multiple factors suggests that widowed older adults are more likely to be dissatisfied with current community-based aging-in-place services. It may be related to the specific characteristics of community home care. Spouses are also considered part of non-professional nursing care, which includes both emotional and practical support (25, 26). Although older adults can have a rich and colorful retirement life in the daytime, the loss of the spouse’s companionship, and the night loneliness will double the widowed older adults. This is confirmed by the analysis of living arrangements, which requires children to pay attention to the psychological changes of widowed older adults and provide them with new companionship (27, 28).

The economic condition of older adults is an influential constraint on their satisfaction with aging in place in the community, which mainly includes occupation and source of income. There is a significant difference in satisfaction between older adults whose source of income is pension/retirement and those who rely on government subsidies for their old age. Government subsidies are preferred by the less well-off older adult, especially those who are unemployed and lack some means of earning a living. Among them, the “five-guarantee households” are the most prominent, and these older adults have no occupation, no spouses, and no children to support them. With the help of state welfare, the “five-guarantee older adult” can enjoy free old-age pensions and medical care in their localities. Therefore, compared with people working in agriculture, forestry, animal husbandry, fisheries, water conservancy, production workers, equipment operators in production and transportation, and other related groups, the satisfaction level of unemployed older adults is higher. This is similar to a survey in the UK, where older adults who own houses pay more for nursing care than those who do not, affecting the enthusiasm of homeowners for nursing care (29). Funding allocation remains a key factor influencing the quality of community-based home care services (29–31).

Community aging, as the community part of the integrated healthcare development model, requires not only the provision of necessary care services but also healthcare services. However, after the analysis of the survey data, it is found that there is no significant difference in the satisfaction evaluation of older adults with different types of medical insurance. Based on the conclusions of other research teams, we find that although community home care can meet some medical needs, some regions and units do not include the combination of medical care institutions in the health insurance reimbursement, and still classify the older adult and medical care fees (32, 33). In addition, the professional health service personnel in the combination of medical and nursing care institutions are uneven, resulting in the community pension can neither fully meet the needs of the older adults’ medical insurance reimbursement, nor provide a professional medical service team, resulting in the situation that the service quality and cost of the combination of medical and nursing care are not fully matched (34, 35), which increases the burden of the older adults in their old age. Once again, the pressure of retirement is focused on economic conditions.

### Suggestion

As a populous province, Shandong Province still shows a large older population in the seventh census, and according to the census results, the size of family households in China is shrinking, and the traditional model of older adults is constantly being impacted. And due to the high-quality pension environment in the coastal areas of Shandong Province, there will be a net inflow of 30,000 older adults in Shandong Province in 2020 (1). These have put forward new requirements for pension services in Shandong Province. Combined with the healthcare integration development model, we still have the following points that require further improvement.

First, we should choose a way of aging that better meets the requirements of older adults according to their emotional and pension demands. With the introduction of various pension methods like tourism pension, ecological pension, intelligent aging, and so on in recent years, the demand for spiritual and cultural consumption of the older population is becoming more and more visible (36–38). We should follow the principle of “old age, old age fun” so that older adults can enjoy a rich and colorful retirement life at the same time, but also get the satisfaction of the emotions, and promote the culture, Tourism, and other industries and the development of cross-fertilization of the pension industry (39) at the same time, to create an innovative and “warm” pension model.

Second, strengthen the policy support and allowances, and actively promote the construction of the aging service system. Due to the number of policy subsidies still a distance from the current cost of old age (40), we should encourage the private capital to join, and establish a pattern of development of the government, private capital, and individual tripartite (41), to promote the continuous development of the health care industry, and at the same time, strengthen the quality of health care service agencies to evaluate and supervise (42), to promote the industry’s benign competition, and to reduce the financial burden of the older adults, and to reduce the cost of old age.

Thirdly, most of the medical and nursing institutions are jointly led by multiple departments, especially those approved and constructed by the Civil Affairs Bureau, the main body of which is for older adults, and the medical part is not included in the scope of medical insurance, which results in the older adults that cannot fully enjoy the treatment of medical insurance reimbursement (43). Therefore, we should be committed to the docking of medical and nursing institutions with medical insurance, and even increase the reimbursement rate of medical insurance, so that we can push forward the health and sustainable development of the medical and nursing institutions through the leverage of the medical insurance policy (44), to establish a healthy working model of “the health department taking the lead in organizing the overall management, the civil administration department providing operational guidance, and the medical insurance department coordinating the policies” (45).

Fourth, according to the disease characteristics of different older adults in different areas, training and selection of professional and unified caregivers to provide professional services in nursing, disease guidance, nutrition, and other aspects of the older adults, so that the older adults can enjoy systematic pension services in the community (46, 47), but at present, China’s health care, nursing, management, and other professionals due to the poor treatment of the pension service units, labor intensity, social status, the brain drain is more serious (48). At present, although relying on the support of national policy, introduced several care service personnel training programs, and the establishment of Guangxi Beihai Recreation and Nursing Vocational College and other professional counterparts of the technical colleges and universities, the training of personnel for a long-term development plan, the near-term benefits of the weak, there is still a need to continue to summarize the base of the construction and personnel training standards and cultivate and recruit talents for nursing homes.

Fifth, based on the different characteristics of the older adult groups, do a better job of the health records of the older adults in the community, through technological empowerment, according to the intelligent terminal data, integration of the service needs of the older adults in each group to provide personalized services to meet the different needs of the older adults, and regular return visits to the service, based on the results of the feedback, and timely adjustment of the personalized service program, to respond to the majority of the older adults’ needs, and to improve the utilization rate of the social resources for the older adults (49).

This study has several notable strengths. First, it spans the period of the COVID-19 pandemic, which exposes the shortcomings of community-based nursing care services during a disease outbreak. Second, it covers a large sample of older adults in Jinan and Qingdao cities of Shandong province, aiming to reflect the current situation of community-based nursing care services. Third, it draws on real-world data from Shandong province and offers suggestions for improving nursing care that are suitable for the local context, providing data support for promoting healthy aging.

This study still has some limitations. First, this study mainly chose Jinan and Qingdao in Shandong Province, which are at a high economic level in Shandong Province and cannot fully reflect the situation in Shandong Province. Second, most of the sixteen communities in this study were concentrated in the cities, with little research in the rural areas. Third, because the survey was conducted during the day of a working day, the older adults with economic pressure and labor ability who went out to work were not interviewed.

## Conclusion

As one of the models of medical and nursing integration, compared with the traditional, pure nursing institutions, community nursing has the advantages of reducing the cost of old age, flexible time, no need to detach from the family, and centralized services (50), and the degree of satisfaction is relatively high among the older population, which is mainly affected by factors such as age, marital status, pre-retirement occupation, the source of income, and the mode of residence. Under the influence of multiple factors, married older adults think that the aging services are average. Agricultural, forestry, animal husbandry, fishery, and water conservancy production personnel and production and transportation equipment operators and related personnel are more inclined to be dissatisfied with the satisfaction level. Given the above factors affecting satisfaction, there is still a need for continuous improvement: increasing attention to the emotional needs of older adults in the context of promoting the development of new ways of aging; reducing the cost of aging; facilitating the seamless integration of health care and aging; strengthening the cultivation of talents in the aging industry; and tailoring personalized aging service solutions.

## Data availability statement

The raw data supporting the conclusions of this article will be made available by the authors, without undue reservation.

## Ethics statement

The studies involving humans were approved by the Life Sciences Ethics Committee of Zhengzhou University (Review Approval No. 2022-07). The studies were conducted in accordance with the local legislation and institutional requirements. The participants provided their written informed consent to participate in this study.

## Author contributions

YW: Data curation, Investigation, Writing – original draft. QZ: Investigation, Writing – original draft. LH: Methodology, Supervision, Writing – original draft. FZ: Conceptualization, Funding acquisition, Methodology, Writing – review & editing.
