# Supplementary material for: Factors related to satisfaction with community-based home aging services in Shandong, China
Source: Front Public Health. 2024 Feb 21;12:1298669. doi: 10.3389/fpubh.2024.1298669 (PMC10916510; doi:10.3389/fpubh.2024.1298669)
Supplement: Supplementary file 1 [file Table_1.DOCX]

Supplementary Table 1 The satisfaction differences among different age groups

| *p-value^1^* | 60-69 years old | 70-79 years old | 80-89 years old | Above 90 years old |
| --- | --- | --- | --- | --- |
| 70-79 years old | 1.000 |  |  |  |
| 80-89 years old | 0.069 | 0.066 |  |  |
| Above 90 years old | 0.165 | 0.149 | 1.000 |  |

^1^ To clarify the intergroup differences in the data, we performed post hoc multiple pairwise tests for the statistically significant data and adjusted the p-values using Bonferroni correction.

Supplementary Table 2 The satisfaction differences among different marital status groups

| *p-value^1^* | Unmarried | Married | Divorced | Divorced |
| --- | --- | --- | --- | --- |
| Married | 0.377 |  |  |  |
| Divorced | 1.000 | 1.000 |  |  |
| Divorced | 0.001 | 0.005 | 1.000 |  |

^1^ To clarify the intergroup differences in the data, we performed post hoc multiple pairwise tests for the statistically significant data and adjusted the p-values using Bonferroni correction.

Supplementary Table 3 The satisfaction differences between pairs of occupational groups before retirement

| *p-value^1^* | Civil servants | Professional and Technical Personnel | Clerical and Related Personnel | Commercial and Service Personnel | Agricultural, Forestry, Animal Husbandry, Fishery and Water Conservancy Producers | Production and Transportation Equipment Operation Related Personnel | The Military Personnel | Other Practitioners | No Occupation |
| --- | --- | --- | --- | --- | --- | --- | --- | --- | --- |
| Professional and Technical Personnel | 1.000 |  |  |  |  |  |  |  |  |
| Clerical and Related Personnel | 1.000 | 1.000 |  |  |  |  |  |  |  |
| Commercial and Service Personnel | 1.000 | 0.675 | 1.000 |  |  |  |  |  |  |
| Agricultural, Forestry, Animal Husbandry, Fishery and Water Conservancy Producers | 1.000 | 0.592 | 1.000 | 1.000 |  |  |  |  |  |
| Production and Transportation Equipment Operation Related Personnel | 0.097 | 0.006 | 1.000 | 1.000 | 0.305 |  |  |  |  |
| The Military Personnel | 1.000 | 1.000 | 1.000 | 1.000 | 1.000 | 1.000 |  |  |  |
| Other Practitioners | 1.000 | 1.000 | 1.000 | 1.000 | 1.000 | 0.100 | 1.000 |  |  |
| No Occupation | 0.905 | 0.022 | 1.000 | 1.000 | 1.000 | 1.000 | 1.000 | 1.000 |  |

^1^ To clarify the intergroup differences in the data, we performed post hoc multiple pairwise tests for the statistically significant data and adjusted the p-values using Bonferroni correction.

Supplementary Table 4 The satisfaction differences between pairs of income source groups

| *p-value^1^* | Retirement Pension/Pension | Children's Subsidies | Financial Support from Relatives and Friends | Labor Income | Government Subsidies | Others |
| --- | --- | --- | --- | --- | --- | --- |
| Children's Subsidies | 1.000 |  |  |  |  |  |
| Financial Support from Relatives and Friends | 1.000 | 1.000 |  |  |  |  |
| Labor Income | 1.000 | 1.000 | 1.000 |  |  |  |
| Government Subsidies | ＜0.001 | 0.001 | 1.000 | 0.915 |  |  |
| Others | 1.000 | 1.000 | 1.000 | 1.000 | 0.462 |  |

^1^ To clarify the intergroup differences in the data, we performed post hoc multiple pairwise tests for the statistically significant data and adjusted the p-values using Bonferroni correction.

Supplementary Table 5 The satisfaction differences between pairs of living arrangement groups

| *p-value^1^* | Living Alone | Living with Spouse/Partner | Living with Children | Living with Spouse and Children | Living with Nanny/Carer | Others |
| --- | --- | --- | --- | --- | --- | --- |
| Living with Spouse/Partner | 1.000 |  |  |  |  |  |
| Living with Children | 1.000 | 1.000 |  |  |  |  |
| Living with Spouse and Children | 1.000 | 0.121 | 1.000 |  |  |  |
| Living with Nanny/Carer | 1.000 | 1.000 | 1.000 | 0.432 |  |  |
| Others | 0.383 | 0.001 | 0.598 | 1.000 | 0.014 |  |

^1^ To clarify the intergroup differences in the data, we performed post hoc multiple pairwise tests for the statistically significant data and adjusted the p-values using Bonferroni correction.
